# “*It’s like nursing a butterfly—so delicate*,* difficult*,* and unpredictable*” – challenges of nurses in caring for patients with epidermolysis bullosa: a qualitative study

**DOI:** 10.1186/s13023-025-03925-8

**Published:** 2025-07-23

**Authors:** Parivash Karimi, Moloud Radfar, Yaser Moradi

**Affiliations:** Patient Safety Research Center, Nursing and Midwifery School, Urmia University of Medical Sciences, Urmia, Iran

**Keywords:** Epidermolysis Bullosa, Nurses, Challenges, Qualitative research

## Abstract

**Background:**

Patients with Epidermolysis Bullosa (EB) face extensive and complex challenges, and nurses play a decisive role in providing care for them and educating their families. However, the multifaceted nature of EB care introduces significant challenges for nurses.

**Objective:**

This study aimed to explore the challenges nurses face in caring for patients with EB.

**Methods:**

This is a qualitative study conducted from August to December 2024. Semi-structured, in-depth, face-to-face interviews were conducted with 15 nurses who provided care to patients with EB in Urmia, Northwest Iran. Data were analyzed using the conventional content analysis approach proposed by Graneheim and Lundman (2004).

**Results:**

Data analysis revealed three main categories of challenges faced by nurses in caring for patients with EB: “*Problematic Parental Reactions*” which includes harmful parental demands, difficult initial encounters, and parental detachment; “*Care Avoidance*” encompassing arduous routine care and the psycho-erosive nature of care; and “*The Forgotten Illness*” characterized by limited organizational support and insufficient organizational knowledge enhancement.

**Conclusion:**

Caring for EB patients presents numerous challenges for nurses, including Problematic Parental Reactions, care avoidance, and the broader context of a forgotten illness. Parental demands, difficult initial encounters, and parental detachment complicate the care, while the demanding and psycho-erosive nature of daily tasks exacerbates nurse fatigue. Furthermore, organizational neglect—stemming from inadequate resources and insufficient training—intensifies these difficulties. These findings highlight the need for targeted training programs and systemic support to address the unique difficulties inherent in caring for patients with EB.

**Supplementary Information:**

The online version contains supplementary material available at 10.1186/s13023-025-03925-8.

## Introduction

Epidermolysis Bullosa (EB) is a rare yet often severe genetic disorder caused by mutations in genes encoding anchoring proteins responsible for stabilizing the basement membrane of the skin and mucous membranes [1, 2]. Characterized by mechanical fragility, blistering, or erosion of the skin, mucous membranes, or epithelial linings of organs in response to minimal trauma, EB can present at varying degrees of severity. Severe forms may exhibit extracutaneous manifestations, such as gastrointestinal, cardiovascular, and genitourinary abnormalities, as well as ocular and oral cavity involvement. These manifestations collectively increase the risk of early mortality [3]. Although symptoms usually appear around birth, lesions may not manifest in some cases until adolescence or later, which can lead to delayed diagnosis in adulthood [4].

According to the National Epidermolysis Bullosa Registry (NEBR) in the United States, the estimated incidence and prevalence of EB are 19.6 and 11.07 per 1 million live births, respectively [5]. Globally, clinical forms of EB affect approximately half a million people, many of whom are children [6]. In Iran, the Burn Research Center of Iran University of Medical Sciences registered data on 538 patients in the EB patient registry system from January to September 2017, indicating a prevalence rate of 6.72 per 1,000,000 in a population of about 80 million [7]. Despite these estimates, many individuals with EB remain hidden due to limited registry systems in most countries and incomplete enrollment in existing registries [5]. Moreover, in Iran, patients with EB are rarely evaluated for disease subtype. In a study by Farokhforghani et al. (2021), only 103 of 538 registered EB patients underwent subtype assessment; dystrophic EB was the most prevalent, representing approximately 75% of these cases [8].

Children with EB face a wide range of challenges, including relentless itching, frequent painful wound care, multiple surgeries, deformities, embarrassment, and difficulties in meeting personal and social needs [9]. Currently, there is no approved cure for EB [6, 10]. Management strategies primarily focus on blister prevention, wound care, pain control, and the early detection and treatment of extracutaneous complications [11].

One particularly distressing aspect for patients with EB is the severe blistering and pain experienced during dressing changes, which can take 2 to 3 h daily. EB can result in multiple wounds at varying stages of healing, some of which may persist long-term. These wounds may lead to complications such as discharge, foul odors, severe blister clusters, deformities, immobility, anemia, malnutrition, infections, and itching [12]. These patients also require multiple surgical interventions due to adhesions in various parts of the body. Such procedures may include the release of fingers or toes, as well as esophageal balloon dilation to alleviate dysphagia. Consequently, they often experience frequent hospitalizations in medical centers and hospitals [13, 14]. Consequently, providing care for patients with EB is exceptionally demanding for nurses [15].

Nurses play a vital role in caring for this population across several areas, including pain management, clinical assessment, wound dressing, patient repositioning, reduction of itching, advising on wound care, ensuring proper nutrition, care planning, and educating family members [16]. Despite their multifaceted responsibilities, many nurses lack the specialized training required for the care of patients with EB, and the number of specialized healthcare centers providing standard care for these patients is limited [17]. As a result, caring for these patients is perceived as both highly demanding and unconventional, which can compromise nurses’ well-being and potentially lead to burnout and other adverse psychological and physical consequences [17].

Research on the specific challenges nurses face in caring for patients with EB is scarce. Given the multidimensional nature of care—often influenced by social, cultural, and religious contexts—a qualitative research approach is particularly effective in exploring nurses’ experiences. By drawing on individuals' lived experiences, qualitative studies provide a deeper understanding of real-world phenomena from the perspectives of those directly involved. One such qualitative research method—content analysis—systematically investigates data to uncover various layers of participants’ experiences, identify patterns, and interpret these findings. Content analysis has been widely employed in healthcare and medical settings to examine interactions between healthcare professionals and patients, as well as healthcare team performance, making it ideally suited to the objectives of this study [18, 19]. For the first time in Iran, this study was conducted to explore the challenges nurses encounter when caring for patients with EB using a qualitative content analysis approach.

## Methods

### Study design

This study was conducted from August to December 2024 using a qualitative approach to explore the experiences of nurses in caring for patients with EB. According to a study by Sandelowski (2010), this approach provides a direct and detailed description of a phenomenon or event [20].

A total of 15 nurses caring for EB patients were selected using purposive sampling. The sample comprised 13 female and 2 male nurses, all working in Urmia, northwestern Iran. To ensure a broad range of experiences, maximum diversity was considered among participants in terms of age, gender, educational level, hospital and ward of employment, and work experience. The study setting included both the nurses’ professional and personal environments. In Urmia, nurses were recruited from two urban university hospitals equipped with more facilities for managing EB. According to statistics from Urmia University of Medical Sciences, 25 patients with EB (16 males, 9 females) are currently alive in west azarbaijan province. Their mean age is 24.08 ± 12.10 years. Several patients died shortly after birth, but reliable data on neonatal mortality are unavailable; nevertheless, nurses also cared for these infants.

### Data collection

Data were collected through face-to-face, semi-structured individual interviews. Participants were initially asked to describe their experiences in caring for EB patients. As the interviews progressed, follow-up probing questions such as “*What do you mean?*“, “*Can you elaborate?*“, “*Could you clarify your point?*“, “*Why?*“, and “*How?*” were used to explore specific aspects of their experiences in greater depth.

All interviews were conducted at times and locations agreed upon by the participants to ensure privacy, minimal distractions, and a comfortable environment. With the participants’ informed consent, each interview was recorded using an audio recorder. Interviews lasted approximately 45 to 60 min. Data collection continued until data saturation was reached, meaning no new concepts emerged in the final three consecutive interviews.

### Data analysis

Data were analyzed using the seven-step content analysis method proposed by Graneheim and Lundman (2004) [21]. After each interview, the recorded audio files were meticulously transcribed by listening to them multiple times. The transcribed data were then typed into a Word file and subsequently imported into MAXQDA 10 (R250412) software for further analysis and categorization. Once the transcripts were thoroughly read, semantic units were identified and coded. These initial codes were then grouped based on similarities and categorized accordingly. Throughout this process, the aim was to maintain maximum homogeneity within categories and maximum heterogeneity between them.

### Rigor

To ensure the validity and reliability of the data, Guba and Lincoln’s trustworthiness criteria—including credibility, confirmability, dependability, and transferability—were applied [22].

Credibility was assessed through prolonged engagement with the data. In other words, it was ensured that sufficient time was devoted to both data collection and analysis. Accordingly, the interview transcripts were independently coded and categorized by the researcher, the primary supervisor, and the consulting professor. The resulting patterns were then compared. In cases where consensus on a particular pattern was not reached, discussions were held among the authors until a mutual agreement was achieved. Additionally, for peer checking, the coded transcripts were returned to four participants to evaluate whether the assigned codes accurately reflected their lived experiences.

For confirmability (or objectivity), the researcher meticulously documented the research process and decision-making procedures to ensure transparency, thereby enabling others to follow and replicate the study. To further establish dependability, external reviewers were engaged to examine the consistency of interpretations between their analyses and those of the researcher, as well as to identify any discrepancies. To accomplish this, both the data and documentation were thoroughly reviewed by an external auditor.

Transferability refers to the extent to which the findings can be applied to other settings or populations. In other words, if individuals from the same or similar groups as the study participants—who were not involved in the research—were to review the findings, they would likely observe parallels between the results and their own experiences. This aspect is partly influenced by the sampling and study design. To assess this, the study findings were shared with non-participating individuals, and the degree of alignment between the results and their personal experiences was evaluated. This alignment evaluation revealed a high level of similarity in this regard [23].

## Results

### Demographic characteristics of participants

In this study, interviews were conducted with 15 nurses caring for patients with EB. The participants held either a bachelor’s or master’s degree in nursing, and their ages ranged from 26 to 54 years (Table 1).

**Table 1:** Descriptive characteristics of the participants

ID	Age (year)	Gender	Ward	Hospital	Educational Level	Work Experience
P1	47	Female	Pediatric Gastroenterology Unit	Pediatric Center	Bachelor	24
P2	42	Female	Neonatal Intensive Care Unit	Pediatric Center	Bachelor	18
P3	43	Female	Neonatal Intensive Care Unit	Pediatric Center	Bachelor	19
P4	38	Female	Pediatric Gastroenterology unit	Pediatric Center	Master	16
P5	32	Female	Neonatal Intensive Care Unit	Pediatric Center	Bachelor	9
P6	31	Female	Infectious Diseases Unit	Infectious Diseases Center	Bachelor	8
P7	28	Female	Pediatric Intensive Care Unit	Pediatric Center	Bachelor	5
P8	34	Female	Neonatal Intensive Care Unit	Pediatric Center	Bachelor	10
P9	32	Male	Emergency Unit	Pediatric Center	Master	7
P10	28	Female	Pediatric Gastroenterology Unit	Pediatric Center	Bachelor	5
P11	52	Female	Emergency Unit	Pediatric Center	Bachelor	28
P12	54	Female	Neonatal Intensive Care Unit	Pediatric Center	Master	31
P13	36	Female	Emergency Unit	Infectious Diseases Center	Bachelor	13
P14	35	Male	Infectious Diseases Unit	Infectious Diseases Center	Bachelor	11
P15	26	Female	Neonatal Intensive Care Unit	Pediatric Center	Bachelor	3

### Categories

Following data analysis, the challenges faced by nurses in caring for EB patients emerged into three main categories: “*Problematic Parental Reactions*”, “*Care Avoidance*”, and “*The Forgotten Illness*” (Table 2).

**Table 2:** Main categories, Sub-Categories, and primary concepts of nurses’ challenges in caring for patients with epidermolysis Bullosa

Categories	Sub-Categories	Primary Concepts
**Problematic Parental Reactions**	Harmful Parental Demands	Superstitious caregiving practices
		Excessive patient visitations
		Parental monopolization of care
		Use of traditional medicine
		Voluntary discharge against medical advice
	Difficult Initial Encounters	Parental shock
		Parental anger over delayed diagnosis
		Distrust in the accuracy of the diagnosis
		Aggressive behavior towards medical staff
		Parental depression
	Parental Detachment	Perceiving the child as a social stigma
		Disruption in parent-child bonding
		Delegating child care responsibilities to third parties
**Care Avoidance**	Arduous Routine Care	Progressive skin fragility
		High risk of injury from touch
		Mucosal vulnerability
		The need for enhanced patient privacy
		Strict sterility requirements
		Difficulties in administering injections
		Fear of disease contagion
	Psycho-Erosive Nature of Care	Disillusionment from less effective care
		Distress from witnessing parental suffering
		Distressing memories arising from working with EB patients
		Guilt over accidental patient injury
		Fear of legal repercussions from parental complaints
		Confronting the fragile emotions of patients
**The Forgotten Illness**	Limited Organizational Support	Lack of specialized EB healthcare infrastructure
		Shortage of trained EB healthcare personnel
		Absence of a comprehensive EB patient association
	Insufficient Organizational Knowledge Enhancement	Insufficient staff training due to the rarity of EB
		Absence of integrated EB-specific care guidelines
		Lack of interdepartmental cooperation

### Category 1: Problematic parental reactions

Based on the nurses’ experiences, *Problematic Parental Reaction*s were one of the most significant caregiving challenges posed by the parents of children with EB. This category comprised three subcategories: *Harmful Parental Demands*, *Difficult Initial Encounters*, and *Parental Detachment*.

#### Sub-Category 1: Harmful parental demands

Families of patients with EB placed specific caregiving demands that were both risky for the patient and challenging for the nurses. These harmful demands included superstitious caregiving practices, excessive visitations, monopolization of care by parents, the use of traditional medicine, and requests for voluntary discharge against medical advice.

In superstitious caregiving, families insisted on using superstitious elements, which in some cases led to skin integrity disruption and infection in their patients. One nurse explained:

*“I have seen families bring small pieces of paper wrapped in plastic or leather*,* claiming they were prayers*,* and place them under the patient’s pillow or blanket. This led to friction on the child’s skin and finally caused wounds. On another occasion*,* a family wanted to sprinkle soil from a religious site onto their child’s wounds*,* believing it would heal them.” (P 12)*.

Some families also insisted on frequent and numerous visitations, which could make it difficult for nurses to enforce infection control measures and prevent unnecessary handling of the child:

*“In one case where we admitted a child*,* visiting hours had just started*,* and both the grandparents came straight to the baby’s bedside. Before I could even get there to tell them not to touch the baby*,* I saw the grandmother already holding and playing with him. This occurred despite repeated instructions to the parents that no one—not even family members—should hold the child at that stage.“(P 8)*.

Moreover, families exhibited a sense of exclusivity in caregiving and therefore insisted on personally taking care of their child in the hospital. One of the participants stated his experience in this regard as follows:

*“The majority of mothers*,* when we explain that their child needs to be treated and cared for by hospital staff and that they should only cooperate and learn rather than assuming full responsibility for care due to the child’s special condition*,* respond by insisting on taking care of the child themselves. This issue has repeatedly led to conflicts with families.” (P 15)*.

Some families attempted to use traditional medicine, which posed additional risks to the patient’s condition.

*“Once*,* a family brought violet oil and insisted on applying it to the child’s skin to reduce redness and blisters. We didn’t allow them to do so. They then insisted on taking the baby to a local hot spring for a bath*,* claiming it would help with healing. We refused again*,* but they didn’t pay attention and said they would definitely do it after discharge.” (P 3)*.

Another major issue was parents seeking voluntary discharge against medical advice, even when the child was in critical condition.

*“Despite their children being in poor condition*,* some families insist on taking them home. This issue has happened multiple times. No matter how much we educate them and tell them it’s dangerous*,* they just refuse.” (P 14)*.

#### Sub-Category 2: Difficult initial encounters

According to the nurses’ experiences, many parents struggled to accept it when they were first informed of their child’s EB diagnosis at birth, which adversely affected the overall care process. The sub-categories in this category include parental shock, anger over delayed diagnosis, distrust in the accuracy of the diagnosis, aggressive behavior toward medical staff, and parental depression.

Parents often experienced an initial state of shock and impaired decision-making:

*“When we first tell them the diagnosis*,* some don’t even know what it is*,* but those who do react really badly. They get petrified and just stare at us. They can’t even talk or make decisions.” (P 15)*.

Some parents expressed anger over why the disease was not diagnosed earlier, despite undergoing prenatal screening and tests.

*“When we inform parents of their child’s condition*,* they get mad at everyone*,* saying*,* ‘We did all the prenatal tests*,* so why didn’t the doctors catch this earlier?’ They act like it’s our fault this happened.” (P 8)*.

A lack of trust in the diagnosis was also frequently observed, with some parents refusing to accept the medical findings.

*“For some parents*,* it takes a long time to accept their child’s diagnosis. Unfortunately*,* they don’t follow medical advice properly. There have even been cases where they still refused to believe the diagnosis even after discharge.” (P 3)*.

In some cases, nurses and doctors faced verbal abuse or aggression from parents upon disclosing the diagnosis. The statement below depicts an experience in this regard:

*“Right after telling parents about their baby’s condition*,* there have been occasions where they’ve started shouting*,* cursing*,* and being completely disrespectful to the staff. This has made both doctors and nurses scared of breaking the news to parents.” (P 15)*.

Following the diagnosis, some parents became depressed and withdrew from caregiving responsibilities. This issue negatively impacted the child’s treatment as mentioned below:

*“After knowing the diagnosis*,* some parents become so emotionally distressed that they isolate themselves. There have been cases where one parent stayed away for days*,* unable to visit their child in the hospital.” (P 12)*.

#### Sub-Category 3: Parental detachment

Based on nurses’ experiences, some parents of children with EB exhibited maladaptive behaviors indicative of parental detachment. This phenomenon encompasses fear of social stigma, disruption of the parent–child bonding, and the delegation of caregiving responsibilities to third parties.

Some families perceived having a child with EB as a source of shame and feared social stigma. These factors could lead them to distance themselves from the child and neglect their parental responsibilities during hospitalization.

*“There were times when the mother or father wouldn’t even come to discharge their child*,* and when we called them*,* they simply said ‘We’re too ashamed to come and take the child home’.” (P 2)*.

A disrupted parent–child bonding was also observed, with some parents avoiding even minimal caregiving responsibilities.

*“Sometimes*,* some mothers barely show any affection when these kids are hospitalized. They don’t even come to talk to them or carefully hug them. This negatively affects the child’s recovery process.”* (P 12).

In some cases, family members other than the parents assumed responsibility for the child’s discharge. Some parents also requested to relinquish their child to social welfare services and did not return to discharge them from the hospital. These issues somehow created ethical and legal dilemmas for the nurses.

*“A few times*,* when it was time for discharge*,* the parents didn’t come*,* and the grandparents showed up instead. No matter how many times we informed the parents*,* they just told us ‘Give the child to the grandmother’. These situations are problematic for us because we can’t be sure whether the grandparents have the physical ability or psychological competence to care for the child.”* (P 4).

*“Once*,* when it was time to discharge a child*,* we kept calling the parents to let them know the kid was ready to go home. But they just said*,* ‘We don’t want the kid*,* give them to social welfare.’ We told them that a child with living parents can’t just be handed over to social services. After a bunch of calls*,* they finally showed up and reluctantly took the kid. A week later*,* the same kid was back*,* in a really bad and critical condition. Turns out*,* the parents hadn’t cared for him at all and had just dropped him off in the courtyard of the social welfare center.” (P 5)*.

### Category 2: Care avoidance

The arduous routine care and the psycho-erosive nature of caring for EB patients led some nurses and healthcare providers to avoid taking on these responsibilities.

#### Sub-Category 1: Arduous routine care

Nurses described EB patient care as particularly burdensome, so that it sometimes leads them to hesitate or refuse to assume responsibility. The defining challenges included progressive skin fragility, a high risk of injury from touch, mucosal vulnerability, the need for enhanced patient privacy, strict sterility requirements, difficulties in administering injections, and fears regarding disease contagion.

One major challenge was the progressive fragility of the patient’s skin, which made it difficult for nurses to observe any improvement despite continuous care.

*“It’s really disheartening when you see a patient getting worse instead of better. Every time I check on them*,* their condition has deteriorated. It makes me not want to work with these patients because they just keep getting worse despite all the routine care.”* (P 1).

Due to the risk of causing harm during routine touch, some nurses refrained from caring for EB patients.

*“It feels awful when I do their routine daily care*,* and then a few hours later*,* I see blisters and wounds exactly where I had touched. It’s like nursing a butterfly—so delicate*,* difficult*,* and unpredictable.” (P 6)*.

The vulnerability of mucosal tissues further discouraged nurses from performing procedures that involved mucosal contact.

*“I remember once there was an EB patient—an older one—who needed an NG tube. But none of the nurses dared to insert it*,* not even the anesthesiologist. How can we be sure we won’t accidentally perforate something inside?”* (P 13).

According to the nurses’ perspectives, although maintaining patient privacy is a standard ethical practice, EB patients required an even higher level of discretion.

*“Whenever we had an EB patient in our ward*,* especially when I worked in the neonatal care unit*,* the parents would like us to refuse any visitations and not disclose the diagnosis. Sometimes*,* distant relatives would come out of curiosity*,* asking questions. While we should respect every patient’s privacy*,* we have to be even more cautious with these patients.”* (P 4).

The strict sterility requirements posed additional challenges, as even standard cleaning procedures had to be performed under sterile conditions.

*“For these patients*,* we have to do everything in a sterile manner because we don’t know if they’ll get an infection even when we perform a procedure cleanly. There have been so many times when*,* despite our best efforts to maintain sterile care*,* their wounds still got infected. It really makes our job tough.” (P 10)*.

Nurses also faced significant difficulties in administering injections and placing IVs, making EB patient care even more challenging.

*“Imagine how hard it is to give these patients injections—it’s like a nightmare. Placing an IV is a huge challenge. We don’t even know how to fix the catheter in place or what to use to secure it. One of the hardest parts of their care is definitely injections and IV access.”* (P 11).

Some nurses even feared that the disease might be contagious, which could lead them to avoid direct care.

*“Maybe my concern sounds ridiculous*,* but how do we know for sure that this disease isn’t contagious? With so little information available*,* who’s to say it isn’t? I have a small child at home*,* and what if my child contracts it or I even catch it myself? Over time*,* I’ve realized that when it comes to diseases*,* nothing is ever completely certain.” (P 14)*.

#### Sub-Category 2: Psycho-erosive nature of care

Experiences such as disillusionment from less effective care, distress from witnessing parental suffering, distressing memories arising from working with EB patients, guilt over accidental patient injury, fear of legal repercussions from parental complaints, and confronting the fragile emotions of patients significantly affected nurses’ psychological well-being. These factors contributed to a tendency among nurses to avoid caring for EB patients.

Nurses often felt disillusioned and disheartened by the limited effectiveness of their care, as they were unable to significantly improve the patients’ wounds. This disillusionment decreased their motivation to provide care for EB patients.

*“It really upsets me when I see that the care I’m providing isn’t making a difference. It honestly shakes my confidence.”* (P 1).

Witnessing the suffering and distress of parents was emotionally exhausting for nurses. To avoid the psychological toll, some nurses preferred not to take on EB patients so they wouldn’t have to interact with the parents.

*“I try not to work on these patients because*,* beyond seeing the child’s devastating condition*,* watching the emotional breakdowns of their parents is heartbreaking. I’d rather take on more cases from other patient groups than deal with this.”* (P 7).

The distressing memories of caring for EB patients lingered long after patient encounters and made it difficult for nurses to move on.

*“Over the years*,* I’ve cared for a few EB patients*,* and whenever I think about them*,* I feel sad. Honestly*,* if a new case comes in*,* I don’t want to work on them because their memories stay with me for a long time and constantly resurface.”* (P 9).

The high likelihood of causing unintentional harm during routine care often left nurses with a deep sense of guilt, which discouraged them from caring for these patients.

*“You want to help the patient get better*,* but then you realize that the areas you handled have blistered and developed new wounds. It’s devastating. I feel guilty and wonder why I even took care of them if I was just going to cause more harm.”* (P 5).

Due to the extreme fragility of EB patients, even routine care could result in injury. Some of the parents who were unaware of the disease’s nature blamed nurses for accidental harm and even filed complaints, and this made nurses hesitant to take on EB patients.

*“Every time I was assigned an EB patient*,* I was scared. I’d talk to my colleagues*,* hoping one of them would take over the case because I was afraid that something might go wrong*,* the patient might get hurt*,* and the parents would sue me. It’s already happened before—there was a case where the parents caused so much trouble for the nurse by blaming her for everything.”* (P 15).

Another significant challenge for nurses was handling the vulnerable and sorrowful emotions of EB patients, especially older children and young adults who were aware of their condition.

*“Some of these patients are old enough to understand what’s happening. Some of them are even young adults. It deeply affects us too when we take care of them and see their pain*,* their fragile emotions*,* and how helpless they feel.”* (P 6).

### Category 2: The forgotten illness

Based on nurses’ experiences, EB was considered a neglected disease by healthcare authorities and organizations. This neglect manifested itself through limited organizational support and insufficient organizational knowledge enhancement.

#### Sub-Category 1: Limited organizational support

Nurses described their experiences with limited organizational support in terms of a lack of specialized EB healthcare infrastructure, shortages of trained EB healthcare personnel, and the absence of a comprehensive EB patient association.

The lack of specialized EB infrastructure forced nurses to rely on non-specialized resources for patient care. This led to increased difficulty in care delivery, patient harm, and wastage of resources.

*“We don’t have dedicated equipment*,* facilities*,* or even a specialized ward for EB patients. We’re not even sure which type of dressings*,* ointments*,* and supplies are best for them. There aren’t any specialized units where we can ensure a more sterile environment*,* so we have to move forward with regular hospital resources. The problem is that this issue not only bothers me as a nurse but prevents the patients from receiving the care they truly need*,* and a lot of supplies like gauze and medical sets are wasted unnecessarily.”* (P 6).

Another major issue rooted in poor organizational support was the shortage of trained staff and the absence of active EB associations in many cities.

*“In our hospital*,* we already have a nursing shortage—that’s a problem on its own. But when one of us is assigned an EB patient*,* we still get the same number of other patients to care for*,* even though EB patients require much more attention. Ideally*,* a nurse caring for an EB patient should have a reduced patient load*,* but when we complain*,* they just say ‘We don’t have enough staff’.”* (P 3).

*“My sister works in Tehran and says there is an EB association here. It’s true that this association sends specialized dressings to registered patients in all cities*,* but it also provides other services that patients in rural areas or smaller towns miss out on due to distance. For example*,* they offer wound care training*,* nutritional guidance for EB patients*,* and even hold events on special occasions that boost morale for both patients and their families. Unfortunately*,* many patients outside of Tehran miss out on these services either because they live too far away or because they are unaware of the registration process.” (P 2)*.

#### Sub-Category 2: Insufficient organizational knowledge enhancement

Another major organizational challenge faced by nurses was the insufficient staff training due to the rarity of EB. Organizations failed to provide proper training on EB, which in turn resulted in a lack of awareness among hospital staff. Nurses identified key issues such as insufficient staff training, the absence of integrated EB-specific care guidelines, and the lack of interdepartmental coordination.

Nurses reported receiving no EB training either during their university studies or in their workplace. As a result, they relied on self-learning through internet sources, which were often incomplete or inconsistent. Treatment approaches remained mostly palliative rather than comprehensive.

*“Not a single person has ever come to our hospital—even though it’s a pediatric specialty center—to train us on EB. No one has explained the symptoms*,* treatment methods*,* or proper care techniques. Even the doctors barely understand this disease. In university*,* they never taught us about it either. So*,* all we do is provide palliative care—apply some ointments and dressings. When we face an EB patient*,* we’re forced to Google everything ourselves just to figure out what we’re dealing with and how to take care of them.”* (P 13).

Another challenge was the lack of an integrated EB care guideline, which meant that nurses had no structured reference material for treating these patients.

*“The treatment and care of EB wounds*,* as well as the ointments and medications used for healing*,* are not integrated. Everyone seems to be doing things based on their own experience. Doctors also don’t follow a uniform protocol—there’s no integrated guideline that we can refer to when an EB patient comes in and say*,* ‘This is the approach we should follow’.” (P 14)*.

Another challenge was the lack of awareness in other hospital departments regarding the sensitivity of EB patients, which often led to unintentional harm during routine medical procedures.

*“When we talk to other departments and tell them ‘These patients are extremely delicate*,* so be extra careful during blood tests*,* X-rays*,* ultrasounds*,* etc.‘*,* they don’t take it seriously. Then*,* you see a simple diagnostic procedure cause so much skin damage that the child suffers terribly.”* (P 2).

## Discussion

In the present study, nurses identified “*Problematic Parental Reactions*”, “*Care Avoidance*”, and “*The Forgotten Illness*” as the challenges in caring for patients with EB.

Harmful parental demands include requests that expose the child to risks such as infections, damage to skin integrity, and worsening of the patient’s condition, thereby increasing the workload for nurses. Parents who insisted on performing superstitious treatments and using traditional medicine for their child, often out of ignorance and cultural beliefs, inadvertently increased the potential harm to their child. The use of traditional medicine for patients, due to the lack of access to complementary medicine specialists and reliance on local healers, resulted in detrimental effects. In a study conducted by Chateau et al. (2023) in South Africa, parents of EB patients turned to traditional and cultural treatments. Their findings emphasized the importance of access to traditional medicine practitioners, fostering discussions to modify viewpoints, and establishing mutual cooperation with these individuals; in their study context, consulting traditional healers was considered the first line of treatment for EB patients. In their study, culture and religion held a special place, and they viewed the situation as a result of the anger of their ancestors or witchcraft [24].

Another identified challenge was the excessive visitation requests from family members, which posed a high risk of infection and harm to patients. This situation can partly be attributed to cultural practices. In Iranian culture, visiting patients is considered commendable. Similarly, in African cultures, it is customary to introduce the newborn child to family members, making it somewhat challenging to restrict interactions [24]. In a study by Coban (2012), isolating burn patients was highlighted as a key factor in reducing infection risk [25]. Conversely, Shao (2022) found that visitation restrictions among hospitalized children can limit family participation in clinical decision-making—an essential component of pediatric care. These restrictions were also shown to increase the family’s desire for early discharge and the child’s return home [26]. Overall, it seems that allowing child visitation only by parents has a beneficial impact on the caregiving process.

In the present study, one of the harmful parental demands challenging nurses was parental monopolization of care and insistence on performing all caregiving tasks for their child. While parental involvement in caregiving activities is essential to foster effective parent-child bonding, proper parental education prior to direct engagement in caregiving responsibilities is mandatory. According to the results of a study by Kristensen and Kronborg (2018), the postnatal period and its associated care play a significant role in shaping the parent-child bond [27]. Another challenge frequently raised by nurses was voluntary discharge against medical advice for various reasons. Alfandre (2009) indicated that leaving the hospital against medical advice could expose the patient to the risk of inadequate treatment, which in turn increases the likelihood of readmission [28]. In a study conducted by Stevens et al. (2016), a different and beneficial approach to caring for EB patients and their families in Australia was examined. Their findings indicated that home nursing programs provided considerable advantages, such as supporting primary caregivers, increasing their comfort, improving work-life balance, enhancing patients’ quality of life, facilitating early diagnosis of squamous cell carcinoma among EB patients, and ultimately reducing hospital visits and workload. Adopting a tailored local model based on these findings could potentially improve the quality of life for patients and families, enhance clinical outcomes, and reduce workload and hospitalization costs [12].

The challenges surrounding nurses’ experiences with problematic parental reactions arise at a time when family-centered care models—originally developed for pediatrics—are receiving renewed emphasis. Nurses strive to base their care delivery on these theories and related models. The foundational idea of this theory traces back to Carl Rogers, who underscored the mutual influence of the treatment process, family dynamics, and individual function and participation in social life. Rogers’ parent-support movement shifted the emphasis from client-centeredness to family-centeredness by highlighting the centrality of family in children’s well-being. According to the family- centeredness theory, family values and practices are broadly implemented in pediatric health, acknowledging family members’ active involvement in addressing medical and psychosocial health issues. Given the necessity of family involvement in child care, alongside the special conditions and required restrictions for these children, nurses involved in this study faced significant challenges [29, 30]. Within this subcategory, issues such as superstitious caregiving practices, excessive patient visitation, parental monopolization of care, and voluntary discharge against medical advice were relatively less explored in the EB literature. By contrast, reliance on traditional medicine is frequently reported.

Another significant challenge mentioned by nurses in this study was the difficulty of initial encounters. In the study by Chateau et al. (2023), parents of EB children experienced emotions such as grief, distress, confusion, anxiety, depression, and emotional turmoil due to having a child with a severe illness [24]. Similarly, Kandel and Merrick (2023) showed that the initial diagnosis of a child’s disability triggered a grief response in parents and other family members [31], which in turn led to reduced parental involvement in caregiving—posing a challenge for nurses. According to Kübler-Ross’s theory, grief typically begins with anger and denial, and progression through subsequent stages requires time [32]. Given that parents often encounter the initial stages of the grief cycle (anger and denial) within the hospital setting shortly after diagnosis, nurses consequently face substantial challenges. Furthermore, Von der Lippe et al. (2017) found that mothers of EB patients experienced a wide range of emotions, including anxiety, confusion, guilt, shock, depression, hopelessness, and emotional detachment from their child [33]. Similarly, issues including parental anger over delayed diagnosis, distrust in the accuracy of diagnosis, and aggressive behaviors toward medical staff were also among the less frequently addressed topics within EB-related literature.

Another challenge mentioned by nurses in this study was parental detachment. Parents created serious difficulties for nurses by distancing themselves from their child, abandoning them, or showing reluctance and lack of participation in caregiving. The stigma associated with illness or disability has been addressed in numerous studies [34, 35]. The stigma surrounding the birth of disabled children can lead to family breakdown, with fathers abandoning their disabled child and leaving the mother solely responsible for caregiving [35, 36]. Chogani et al. (2021) revealed that fathers were often absent and exhibited emotional detachment from their children, while the caregiving burden was primarily borne by the mother [37]. Chateau et al. (2023) also found that parental reactions varied—some adapted and showed hope in supporting their child, while others responded with anger or even rejection [24]. Additionally, Muddle et al. (2022) found that when a child is unable to establish a normal emotional bond with their caregiver, it negatively affects the parent-child attachment [38]. Findler et al. (2016) concluded that when parents feel overwhelmed by the demands of caring for a disabled child and lack sufficient support, they struggle to form secure bonds with their child [39]. Coughlin and Sethares (2017) also found that parents may experience recurrent grief over their child’s life circumstances, which can lead to detrimental effects on parent-child relationships, insecure attachments, lack of intimacy, and resentment [40]. In the study by Von der Lippe et al. (2017), mothers of EB patients experienced emotional detachment from their child [33]. According to Kathryn Barnard’s theory, healthy child development relies upon caregivers responding lovingly and consistently to infant signals. Furthermore, individual and physical characteristics of both caregiver and infant significantly influence the parent-infant system [41]. Some parents of children with EB struggle to establish healthy parent-infant relationships due to their child’s distinct physical appearance and may refuse caregiving responsibilities. Issues such as perceiving affected children as social stigmas and delegating their care to third parties are also infrequently discussed; previous studies have focused mainly on disruptions to bonding. Parents of EB patients should receive counseling, education, and psychological and spiritual support to better understand their child’s condition, manage their requests effectively, learn appropriate caregiving methods, and cope with their diagnosis. These interventions would ultimately improve parent-child relationships and reduce the challenges faced by nurses in providing care.

The arduous nature of routine care and the psychologically erosive aspects of caregiving contribute to care avoidance among nurses treating patients with EB. These routines are not only physically demanding but also exert both short- and long-term psychological effects on nurses. Chateau et al. (2023) reported that healthcare personnel often struggle to separate personal emotions, maintain neutrality, and overcome maternal instincts. Some nurses expressed fears regarding disease progression, pain management, and causing blisters or additional injuries during patient contact. During consultations and dressing changes, emotions such as grief, fear, and distress were especially pronounced. Moreover, witnessing both the physical suffering of patients and the emotional distress of their parents proved profoundly upsetting. Feelings of inadequacy in providing care and additional pressure from parents further contributed to disillusionment and emotional exhaustion [24]. Novel concerns identified in this study—including need for enhanced patient privacy and nurses’ fear of disease contagion —are seldom addressed in prior EB work. Although nurses know EB is genetic, apprehension about contagion persists.

Dures et al. (2010) corroborate the *Psycho-Erosive Nature of Care* category, where nurses reported guilt and helplessness due to the transient effectiveness of treatments [17]. Similarly, Martin et al. (2019) support the *Care Avoidance* category by highlighting varied reactions among healthcare workers toward EB patients; while some adapted, others resorted to avoidance mechanisms or experienced a sense of powerlessness [42].

In addition, nurses reported distress at witnessing parental suffering and fear of legal repercussions from parental complaints—topics rarely explored in EB-specific research. Nurses perceive caring for EB patients as a significant stressor that places them under both physical and psychological pressure, often leading them to avoid patient care responsibilities. According to Lazarus and Folkman’s Psychological Stress and Coping Theory, the avoidance coping strategy involves bypassing or evading stressful situations rather than directly confronting and resolving them. However, prolonged reliance on avoidance coping is discouraged, as it detaches individuals from the problem, thus preventing the development of adaptive behaviors [43].

Caring for EB patients is particularly challenging; nurses require robust organizational support in the form of comprehensive disease education, access to psychological services, financial incentives, and paid leave to safeguard their emotional well-being. Furthermore, nurses must acquire sufficient motivation, knowledge, and psychomotor preparedness to deliver optimal care to these patients.

Insufficient organizational support and inadequate investment in EB-related knowledge contribute to its status as a “*Forgotten Illness*”. Despite these gaps, nurses are expected to deliver high-quality care even when resources and training are limited. Findings of the study by Chateau et al. (2023) align with those of ours in terms of limited organizational support, wherein caregivers voiced concerns about the lack of specialized EB training and the restriction of care largely to wound and pain management. They further noted the absence of specialized dermatology units, inadequate facilities, and prohibitively high dressing costs as major obstacles [24]. Martin et al. (2019) underscored the need for research aimed at developing more accurate and rapid EB diagnostic methods, as well as enhanced training to facilitate timely diagnosis and referral [42]. Dures et al. (2010) likewise emphasized inadequate organizational support. They revealed a scarcity of trained EB specialists and limited awareness among many healthcare professionals regarding the complexity of the disease, patient needs, and the psychological toll on families [17]. Martin et al. (2019) also support the insufficient organizational knowledge enhancement by highlighting education for healthcare workers as vital to providing both physical and psychological care for EB patients and their families [42]. In a similar vein, El Hachem (2014) stressed the importance of interdisciplinary collaboration for improved EB patient management [10].

In many countries, organizations such as DEBRA provide direct services to EB patients and families, supply educational materials, and collaborate with healthcare and research institutions to bring substantial improvements to EB patients’ lives. Additionally, DEBRA has established nurse education programs specifically designed to address nurses’ training needs, facilitate discussions, and respond to their inquiries [44]. However, Iran is not among the countries currently receiving support from DEBRA. Furthermore, DEBRA actively develops clinical practice guidelines for healthcare professionals, patients, and their families globally. These evidence-based guidelines comprehensively address various aspects of EB management, including skin and wound care, emergency care, and ocular care [45]. In contrast, healthcare providers in Iran have limited awareness of these guidelines, and the lack of Persian translations further restricts their usage. It is recommended that Health-care managers should seek educational, research, and dressing-material support from DEBRA and similar organizations.

Nonetheless, this study found that many hospital departments lack sufficient EB-related knowledge, which in turn can exacerbate the challenges nurses face. This study also highlights the paucity of intra-hospital collaboration, attributable largely to inadequate staff training and awareness—an issue seldom examined previously. Therefore, healthcare organizations must take the initiative to develop the necessary infrastructure, procure appropriate equipment, and establish specialized units capable of providing care for EB patients. Additionally, they should enhance the knowledge and expertise of their healthcare workforce to improve the management of EB patients. Furthermore, by raising public awareness about EB and its challenges, they can create opportunities for the establishment of EB-focused charitable organizations. Simultaneously, they should integrate EB education across all levels of healthcare organizations and develop standardized educational guidelines for EB care. International collaborations with organizations such as DEBRA, which provide both financial and educational support to EB patients and healthcare providers, could be highly beneficial.

### Limitations & strengths

One limitation of this study involved building trust with nurses, who initially expressed hesitancy regarding discussing organizational constraints. Trust-building was facilitated by clearly explaining the research objectives, providing documentation and authorizations, and ensuring anonymity for participants. Another limitation was the study’s geographical restriction to northwest Iran. Given the country’s significant size and cultural and ethnic diversity—factors potentially influencing nurses’, patients’, and families’ behaviors—it may not be possible to generalize the findings to other regions, although generalizability is typically not the primary aim of qualitative research.

A strength of the present study lies in its pioneering role in elucidating the challenges faced by Iranian nurses working with EB patients, providing novel, firsthand insights into these experiences. Nonetheless, further studies conducted in other Iranian cities are necessary for a more comprehensive understanding of these nursing challenges.

## Conclusion

Despite the rarity of EB and the relatively small number of affected individuals, caring for even a limited number of these patients poses significant challenges for nurses. These challenges may stem from parents, manifesting as *Problematic Parental Reactions*, from the inherently difficult nature of care, which leads to *Care Avoidance*, and from healthcare organizations, where EB becomes a “*Forgotten Illness*”. Some of the findings of the present study were original and had been less addressed in the EB literature, although some of these concepts have been explored in studies examining children with conditions other than EB. For instance, in the context of problematic parental reactions, issues such as superstitious caregiving practices, excessive patient visitation, parental monopolization of care, voluntary discharge against medical advice, parental anger over delayed diagnosis, distrust in the accuracy of the diagnosis, aggressive behaviors towards medical staff, as well as perceiving the child as a social stigma and delegating childcare responsibilities to third parties were among the topics that received limited attention in the EB literature. In terms of care avoidance, issues such as the need for enhanced patient privacy, staff fears regarding potential disease transmission, distress from witnessing parental suffering, and fear of legal repercussions from parental complaints were also less frequently discussed in the EB literature. Regarding the concept of “The Forgotten Illness,” issues such as the lack of collaboration among hospital units due to insufficient knowledge enhancement were among the topics that had been less addressed in previous studies.

### Implications

The findings of the present study offer nurses clear insights into potential challenges associated with caring for patients diagnosed with EB. This study has elucidated the challenges nurses encounter when interacting with the families of EB patients. By becoming aware of these challenges, nurses can better prepare themselves for managing interactions with patients and their families, thus responding appropriately to emotional or potentially harmful family behaviors. Consequently, this preparedness enables optimal patient care, assists families in adapting to their child’s condition, and promotes healthier parent-child relationships. Families of EB patients should receive targeted educational interventions and psychotherapy to better manage their emotional responses and problematic behaviors, thereby minimizing the challenges faced by nurses.

Many challenges originate from insufficient knowledge and training, which place heavy physical and psychological demands on nurses. Incorporating specialized EB content—including disease management and care protocols—into undergraduate curricula and in-service programs is therefore essential. Additionally, nurses require comprehensive organizational, financial, and psychological support to effectively manage the demanding care routines of EB patients, cope constructively with the emotionally taxing nature of this disease, and ultimately reduce their inclination toward care avoidance.

Furthermore, the present study underscores the indispensable role of organizational support and highlights the negative outcomes associated with insufficient organizational knowledge enhancement. Emphasis should be placed on staff training to enhance their collective knowledge about EB, governmental support, establishment of charitable governmental and non-governmental organizations, and collaboration with institutions like DEBRA to better address the needs of EB patients.

## Electronic supplementary material

Below is the link to the electronic supplementary material.

Supplementary Material 1

## Data Availability

The data that support the findings of this study are available from the corresponding author upon reasonable request.

## Abbreviations

EB: Epidermolysis Bullosa
NEBR: National Epidermolysis Bullosa Registry
IV: Intra Venous
NGT: Naso Gastric Tube
