# Supplementary material for: “It’s like nursing a butterfly—so delicate, difficult, and unpredictable” – challenges of nurses in caring for patients with epidermolysis bullosa: a qualitative study
Source: Orphanet J Rare Dis. 2025 Jul 23;20:371. doi: 10.1186/s13023-025-03925-8 (PMC12285049; doi:10.1186/s13023-025-03925-8)
Supplement: Supplementary file 1 — Supplementary Material 1 [file 13023_2025_3925_MOESM1_ESM.docx]

| # | Comments | Responses to the reviewers |
| --- | --- | --- |
|  | Reviewer 1 | |
| 1 | Reviewer #1: Qualitative study presenting a relevant and original analysis of the challenges faced by nurses in caring for patients with epidermolysis bullosa (EB), a rare and debilitating condition.  Detailed critical analysis of the main aspects of the manuscript: Relevance and Originality - Relevance: The study highlights the complexity of caring for patients with epidermolysis bullosa (EB) and the need for efficient organizational support. The research reveals deficiencies in the training and working conditions of nurses, proposing improvements for the well-being of these professionals and, consequently, for the quality of patient care. - Originality: A pioneering study carried out in Iran, the specific cultural approach enriches the global literature, offering new perspectives on the difficulties faced in under-explored contexts. | **Thank you for your valuable comments. Your insights are encouraging and highly appreciated.** |
| 2 | Methodology  - The qualitative approach is appropriate for exploring the lived experiences of nurses, allowing an in-depth understanding of the challenges they face.  - The purposive selection of 15 nurses (13 women and 2 men) offers diversity in terms of age, gender, education and professional experience. However, the sample size can be considered small for broader generalizations, which limits the transferability of the results.  - The semi-structured interviews were well conducted, using open-ended questions that encouraged participants to share their experiences in detail. Data saturation was achieved, ensuring validity.  - Content analysis based on Graneheim and Lundman was applied rigorously. Although it does not involve traditional statistical analysis due to the qualitative nature of the study, the method used is suitable for identifying patterns in the experiences reported.  - Ethics: The study followed sound ethical principles, including informed consent and protection of participants' privacy. | Thank you for your comment. Regarding sample size, the number was determined based on data saturation, achieved when no new concepts emerged from the last three interviews. |
| 3 | Results  - Clarity and organization: The results are presented in a clear and structured way in three main categories:  1. Problematic Parental Reactions, which include harmful parental demands, difficult initial encounters and parental distancing.  2. Care Avoidance, which addresses the arduous and psycho-erosive nature of daily routines.  3. The Forgotten Illness, which highlights limited organizational support and lack of adequate training.  - Relevance: The findings are directly relevant to the research question, highlighting practical and emotional barriers faced by nurses.  - Consistency: The results are in line with the methodology adopted and corroborate previous studies on the challenges of rare disease care. | Thank you for your valuable comments. Your insights are encouraging and highly appreciated. |
| 4 | Discussion  - The interpretation is appropriate and well-founded. The authors connect the results to the practical needs of specific training for nurses and the organizational support required.  - Limitations are acknowledged by the authors, including the small sample size and the focus on a single geographical region (Urmia, Iran). These limitations restrict the generalizability of the results to other cultural or population contexts.  - The article discusses important implications for clinical practice, such as the urgent need for specific training programs for nurses dealing with EB.  - The study fills gaps in the literature by addressing the challenges faced by nurses in a specific cultural context. | We appreciate your insightful comment. Indeed, generalizability in qualitative research differs from quantitative studies. In qualitative research, generalizability is limited and addressed under the concept of transferability, as elaborated in the rigor section. |
| 5 | Quality of Writing - The text is clear, direct and accessible even to readers outside the immediate context of the research. - The article follows a well-defined logical structure (introduction, methods, results, discussion), making it easy to read. - No significant grammatical or spelling errors were identified in the manuscript. - The references are complete, accurate and relevant, adequately supporting the claims made in the text. Ethics and Conduct - There is no explicit mention of conflicts of interest; it would be important to include this information for greater transparency. - The article appears to be free of plagiarism; the data presented is original and clearly derived from the interviews conducted. - There is no evidence of manipulation or inconsistencies in the data presented. Compliance with Guidelines - The manuscript is in line with the general guidelines of the Orphanet Journal of Rare Diseases, including proper structuring of the text (introduction, methods, results and discussion). | Thank you for your constructive remark. The conflicts of interest are provided under the heading “Competing interests” in the manuscript. |
|  | Reviewer 2 | |
| 1 | Reviewer #2: This is an interesting study on the important topic. EB is a such a devastating disease and impact on health professionals dealing with this disease is often overlooked. Thee are some suggestions that could be applied to improve the quality of the manuscript or the way the data is presented.  The methodology and process followed in analysis is fine. The finding support the general consensus in the field however authors could better highlight the novelty of the findings or present key take away messages as bullet points at the end. Alternatively following should also be addressed: | Thank you for your valuable feedback. The findings that appeared novel to the authors and less frequently addressed in previous literature were highlighted following the discussion of each subcategory, and summarized again in the conclusion. |
| 2 | 1. Authors need to acknowledge different types and severity of EB, as impact on nurses caring for milder patients would not be same as reported. Authors also need to acknowledge that certain subtypes are worse at birth and get progressively better while others are better at birth and get progressively more severe as children age, so stage at which patient is e.g., severe newborn, vs palliative care patient will carry different effects on the nursing staff and their interactions with family. | Given the novelty and underdevelopment of EB registries in Iran, determining EB subtypes is frequently impossible. Many families and patients, especially adults living with EB, are unaware of their specific subtype. Furthermore, physicians, nurses, and other healthcare staff often experience challenges even diagnosing EB itself, let alone distinguishing subtypes. Nurses included in our study cared for patients from all subtypes admitted to their hospitals, and thus their experiences are reported collectively. This clarification was added to the Introduction to highlight that EB subtyping is often unestablished in Iran. |
| 3 | 2. Cultural aspect needs to be better highlighted as relationships between health care staff and patients or families might carry different annotations for example Western culture vs Asian culture. | Issues identified as culturally influenced were incorporated and comparatively discussed with African cultural contexts. |
| 4 | 3. The level of distress experienced is also dependent on healthcare provisions in different countries, authors briefly mention access to advanced dressings, but this could be expanded to cover other areas of support that families may access or have a lack of access to in the community, for example in-home nursing, allied health, social support, financial government assistance etc. as this will affect how family experiences EB and in fact how they project then in their interactions with health professionals. | Thank you for your insightful suggestion. Since the current study focused on nurses working in hospital settings, parental anger and distrust regarding delayed or uncertain diagnosis were highlighted prominently. Nurses also emphasized minimal governmental, social, and financial support provided to EB patients and their families. However, this primarily constitutes a challenge for patients and their families, whereas organizational support-related challenges faced by nurses were addressed separately under the category "The Forgotten Illness." |
| 5 | 4. The report focused on experience of nurses from one same hospital? Form provincial city? Please be clear on this, and state if experience might be different for families and health professionals working in rural vs metropolitan areas. | Nurses were selected from two university-affiliated hospitals in the city, serving as pediatric and infectious disease referral centers frequently attended by EB patients. The column identifying each hospital was added to the manuscript. |
| 6 | 5. Reference 11 Stevens et al., 2016 highlighted the benefits of different approaches to models of care in EB, evaluating effects of in home nursing vs hospital nursing and emphasizing benefits of seeing these patients in home environment, this could be more discussed in the discussion or highlighted as potential areas for improving the models of service delivery to decrease the challenges or impacts on both families and nurses who are providing the care. | We appreciate your recommendation of the relevant article; pertinent information from this suggested reference has been incorporated into the manuscript discussion. |
| 7 | . In study design section, there should be comment on environment in which care is provided, i.e. single tertiary hospital? How many EB patients are seen in this centre annually? What is the patient basic demonographic in terms of gender, age, EB type? | Information regarding the number, gender, and mean age of living EB patients was updated based on data acquired from the Special Diseases Unit of Urmia University of Medical Sciences. However, due to insufficient EB subtype diagnostics within Iran, these details remain largely unavailable. |
| 8 | Table 2 and description of subcategories it would be good to get a sense of how frequent some of these experiences were. Especially since nurses ranged in years of work from 3-24 yrs. In the discussion authors could discuss which of the categories highlighted were expected based on literature and what is know about EB and which ones were novel - for example disruption of patent-child bond is very often reported as is the psychosocial and physical impact on EB and family. | Following each subcategory discussion, findings identified as novel or infrequently addressed in previous EB literature were clearly delineated. |
| 9 | 8. I would re-consider if quote relating to disease being contagious should be included or not as its alarming that the nurse would not understand the difference between contagious or genetic condition. Although this supports need for better education on the topic. | We appreciate your observation. Unfortunately, some nurses, despite basic awareness that EB is a genetic condition, expressed persistent concerns about disease contagiousness. This aspect has been emphasized in the discussion. |
| 10 | 9. Difference in hospital resources and care systems should be acknowledged, for example in Western hospitals there would often be a designated Complex care nurse for cases like EB, which would have higher level of training and reduced patient load due to complex care required, but this may not be a case in Iranian hospital impacting the nurse experience. Some wealthier countries like Australia have EB nurses in hospitals (paid for by DEBRA patient charity) to support EB patients. This could be discussed. | We value your remark. The role and supportive activities of institutions such as DEBRA were described, and comparisons between such support mechanisms internationally and within Iran were highlighted. |
| 11 | 10. Comments about lack of or lack of knowledge of EB specific care guidelines are concerning. Many EB specific clinical practice guidelines are available freely to download form DEBRA International website - both ones for health professionals and patients, and some translated to multiple languages. This should be acknowledged noting that knowledge of these is yet to be more broadly disseminated and more language translations required to reach those in more developing counties where care and resources are not optimal. Please note there is also a guideline for wound care in countries where expensive dressings are not available offering nurses options for provision of good quality care. | We appreciate your comment. The discussion specifically addressed the importance and role of DEBRA's educational programs for healthcare providers. |
| 12 | 11. Conclusion could better highlight the novelty and next research steps as well as discuss the limitations of the current study. | The Conclusion has been enriched, explicitly emphasizing novel findings. Furthermore, strengths, limitations, and recommendations for future research have been presented in a dedicated subsection. |
|  | Reviewer 3 | |
| 1 | Strengths of the Manuscript:  1. Relevant and Timely Topic: The study addresses a critical yet underexplored area in nursing practice, focusing on the unique challenges faced by nurses caring for patients with Epidermolysis Bullosa (EB), which is essential for improving patient outcomes and optimizing healthcare protocols. This is a valuable contribution to both the fields of nursing and rare disease care. The focus on qualitative research enriches understanding of healthcare providers' real-world challenges, contributing valuable perspectives for nursing education and policy development.  2. Methodological Rigor: The study employs a qualitative design with in-depth, semi-structured interviews, which is appropriate for capturing the nuanced experiences of nurses in this context. The use of purposive sampling ensures that diverse nursing experiences are included. The use of Graneheim and Lundman's conventional content analysis provides a structured approach to data interpretation.  3. Rich, Contextual Data: The manuscript effectively presents direct quotes from participants, capturing the emotional and physical toll of caring for EB patients. This enhances the credibility and authenticity of the  4. Clear Organization of Themes: The manuscript presents well-defined categories of challenges. The identification of three major categories (Problematic Parental Reactions, Care Avoidance, and The Forgotten Illness) provides a systematic understanding of the psychosocial and institutional barriers affecting nursing care for EB patients. The study appropriately contextualizes its findings by integrating supporting literature from previous works, including those related to caregiver burden, patient stigmatization, and organizational neglect. | We sincerely thank you for acknowledging the manuscript’s strengths and providing thoughtful feedback. |
| 2 | Major Points for Revision:  1. Manuscript Format (Letter to the Editor):  The current structure, at over 15,000 words for a mainly descriptive work, makes difficult to interpret scientifically and makes it very difficult to follow. The authors should significantly condense the manuscript to focus on the most impactful findings and critical reflections. I would recommend for publication following major revision as a Letter to the Editor. | Thank you for your valuable comments. The manuscript is approximately 9,400 words, a length typical in qualitative studies due to direct quotations, explanations of quotes, and extended discussion sections reflecting numerous extracted concepts. The current version represents a condensed form, intentionally excluding additional redundant quotes to ensure each quotation uniquely conveys distinct concepts. The study was not designed as a Letter to the Editor, as the complexity and breadth of identified nursing challenges in Iran warranted comprehensive exploration. However, your suggestion is valuable and will be considered should new nursing challenges regarding EB emerge in the future, possibly warranting publication in a letter format. |
| 3 | 2. Lack of Strong Theoretical Framework:  While the study employs content analysis, it would benefit from a more explicit theoretical foundation (e.g., stress and coping models, nursing theories, or patient-centered care frameworks). The authors should integrate nursing theory or psychosocial models to anchor their findings in established literature. | In interpreting our findings, relevant theories such as Family-Centered Care theory rooted in Carl Rogers’s work, Kubler-Ross’s Grief Theory, Kathryn Barnard’s Parent-Child Interaction Theory, and Lazarus and Folkman’s Psychological Stress and Coping Theory were explicitly incorporated into the manuscript. |
| 4 | 3. Focus and Thematic Tightening:  The three primary themes, while comprehensive, may be too broad for a concise letter format. It would be beneficial to focus on the most striking insights, such as Problematic Parental Reactions and Care Avoidance, which are likely the most compelling to the readership. | We fully agree with your observation. More prominent and novel issues less frequently explored in specialized EB literature were highlighted after discussing each subcategory, particularly regarding parental reactions and care avoidance. Additionally, a summary of these novel findings has been provided in the conclusion section. |
| 5 | 4. Sample Size and Generalizability Concerns:  The study is limited to 15 nurses in Urmia, Iran, which restricts generalizability. The authors should acknowledge these limitations more explicitly and discuss how findings may (or may not) translate across different healthcare settings. | We agree with your insightful remark; as a qualitative study, our findings inherently possess limited generalizability, which is not the primary objective of qualitative research. A dedicated subsection addressing this issue has been added. Sample size determination was based on data saturation, as indicated by no new concepts emerging from the last three interviews, resulting in a sample of 15 nurses, a common practice in qualitative studies. |
| 6 | 5. Clarity in Clinical Implications: The manuscript should clearly articulate the direct implications of these findings for clinical practice, including specific recommendations for nurse training, organizational support, and patient-family communication strategies. | Thank you for identifying this gap in our manuscript. Accordingly, implications related to enhancing nurses' awareness about challenges in EB patient care, nursing education on EB management, and emphasizing organizational support have been incorporated. |
| 7 | 6. Citation and Context:  The manuscript includes extensive literature references, but these should be selectively reduced to a few key citations that directly support the core message of the letter, aligning with concise referencing style. | We appreciate your valuable comment. Due to the extensive scope of nursing challenges identified and comprehensive literature reviewed, numerous studies were discussed to substantiate our findings. |
| 8 | 7. Language and Tone:  The tone should be adjusted to fit the concise, evidence-focused style. This includes removing overly emotional phrasing and focusing on actionable insights. | We acknowledge your point regarding the emotional tone of certain quotations. However, verbatim quotations must be preserved to maintain authenticity and accurately reflect participants' experiences. |
| 9 | Specific Areas for Improvement:  * Abstract and Introduction:  These sections should be condensed into a single paragraph that succinctly captures the study's purpose, methods, key findings, and implications. | Since the manuscript was not intended as a Letter to the Editor, its abstract contains 243 words, well within the journal’s maximum limit of 350 words. Following your suggestion, implications have been clearly presented after the Conclusion section. |
| 10 | * Results Presentation:  While the quotes are impactful, the number should be reduced to only the most illustrative examples to maintain a concise tone. | Our writing process emphasized conciseness and clarity in presenting results. Multiple revisions by the authors involved removing redundant quotations and streamlining others to eliminate nonessential text. |
| 11 | * Conclusion:  The conclusion should directly address potential interventions and strategies for supporting nurses, rather than broadly summarizing the challenges. | Interventions supporting nursing staff were explicitly mentioned in both the Conclusion and Implications sections, as per your recommendation. |
| 12 | * Consider restructuring the manuscript as a focused, reflective piece that highlights the emotional and organizational burdens faced by nurses in this context, while offering clear, evidence-based recommendations for improvement. | Recommendations within the Discussion and Conclusion sections were expanded for clarity and comprehensiveness, and subsequently summarized within the Implications section. |
| 13 | * This study has strong potential for publication but requires major revisions to improve methodological clarity, theoretical grounding, and discussions on ethical challenges and generalizability. With these enhancements, it could make a valuable contribution to the field of nursing care for rare diseases. | We sincerely appreciate your constructive comments, resulting in substantial improvements to both the Methodology and Discussion sections, significantly enhancing the quality and depth of our manuscript. |
